# 4-Hydroxy­meth­yl-2-methoxy­phenol

**DOI:** 10.1107/S1600536809043025

**Published:** 2009-10-23

**Authors:** Qiang Wang, Suo-Ping Li

**Affiliations:** a Henan University, Kaifeng 475004, People’s Republic of China

## Abstract

The title compound, C_8_H_10_O_3_, is close to planar (r.m.s. deviation = 0.042 Å) apart from the hydroxyl O atom [deviation = 1.285 (1) Å] and an intra­molecular O—H⋯O hydrogen bond occurs. In the crystal, inter­molecular O—H⋯O links lead to chains propagating in [001].

## Related literature

For a related compound used as a food additive, see: Kumar *et al.* (2004 ▶); Shaughnessy *et al.* (2001 ▶).
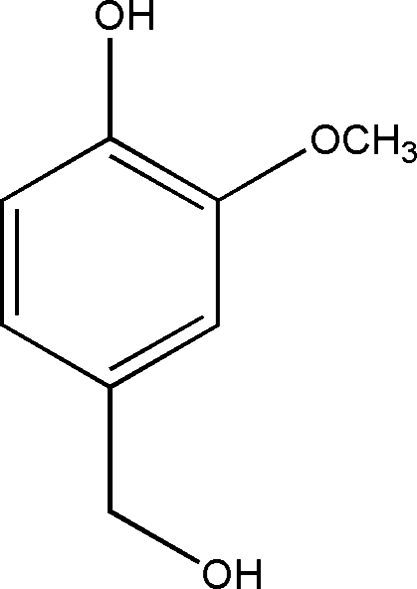


## Experimental

### 

#### Crystal data

C_8_H_10_O_3_
*M* *_r_* = 154.16
Monoclinic, 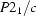

*a* = 9.8476 (6) Å
*b* = 6.1721 (4) Å
*c* = 15.4915 (7) Å
β = 126.877 (2)°
*V* = 753.19 (8) Å^3^
*Z* = 4
Mo *K*α radiation
μ = 0.10 mm^−1^
*T* = 293 K
0.29 × 0.11 × 0.07 mm

#### Data collection

Bruker SMART CCD diffractometer
Absorption correction: multi-scan (*SADABS*; Bruker, 2001 ▶) *T*_min_ = 0.971, *T*_max_ = 0.993
3996 measured reflections
1475 independent reflections
1249 reflections with *I* > 2σ(*I*)
*R*_int_ = 0.015

#### Refinement

*R*[*F* ^2^ > 2σ(*F* ^2^)] = 0.037
*wR*(*F* ^2^) = 0.102
*S* = 1.05
1475 reflections
102 parameters
2 restraints
H-atom parameters constrained
Δρ_max_ = 0.24 e Å^−3^
Δρ_min_ = −0.19 e Å^−3^

### 

Data collection: *SMART* (Bruker, 2001 ▶); cell refinement: *SAINT-Plus* (Bruker, 2001 ▶); data reduction: *SAINT-Plus*; program(s) used to solve structure: *SHELXS97* (Sheldrick, 2008 ▶); program(s) used to refine structure: *SHELXL97* (Sheldrick, 2008 ▶); molecular graphics: *PLATON* (Spek, 2009 ▶); software used to prepare material for publication: *PLATON*.

## Supplementary Material

Crystal structure: contains datablocks global, I. DOI: 10.1107/S1600536809043025/hb5154sup1.cif

Structure factors: contains datablocks I. DOI: 10.1107/S1600536809043025/hb5154Isup2.hkl

Additional supplementary materials:  crystallographic information; 3D view; checkCIF report

## Figures and Tables

**Table 1:** Hydrogen-bond geometry (Å, °)

*D*—H⋯*A*	*D*—H	H⋯*A*	*D*⋯*A*	*D*—H⋯*A*
O1—H1*A*⋯O2	0.82	2.31	2.6669 (16)	107
O1—H1*A*⋯O3^i^	0.82	1.96	2.7390 (16)	158
O3—H3*B*⋯O1^ii^	0.84	2.07	2.8666 (15)	158

Symmetry codes: (i) 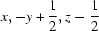; (ii) 
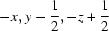
.

**Fig. 1.**
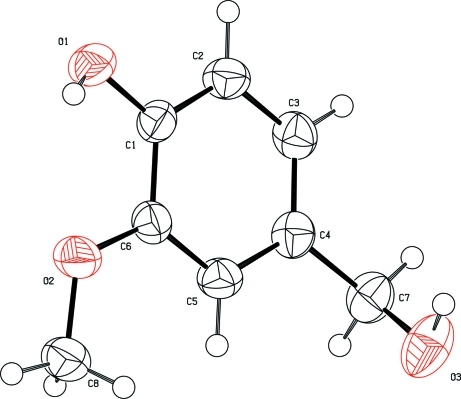
The molecular structure of (I), showing displacement ellipsoids drawn at the 50% probability level.

**Fig. 2.**
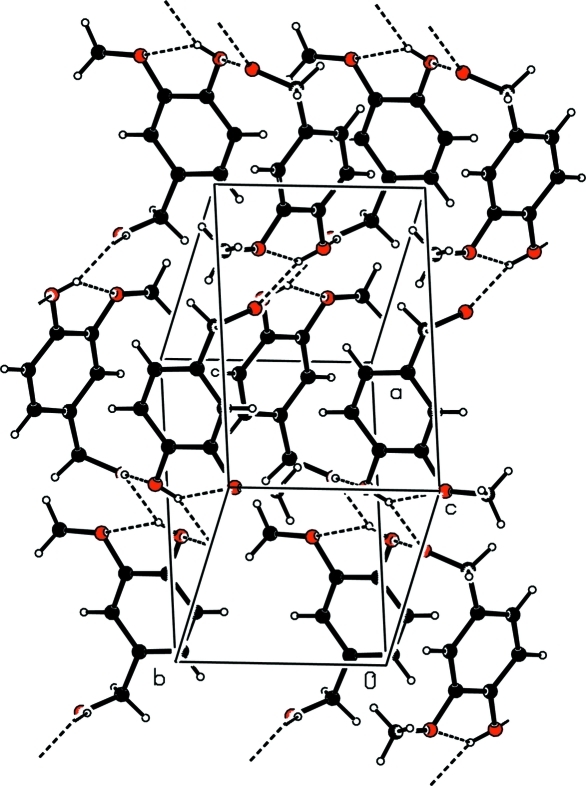
One-dimensional structure of (I) showing hydrogen bonds as dashed lines.

## supplementary crystallographic information

### Comment

4-hydroxy-3-methoxybenzaldehyde is one of the commonly used food additives. In recent years, it was discovered that 4-hydroxy-3-methoxybenzaldehyde has anti-oxidation (Kumar *et al.*, 2004) and inhibition activity of gene mutation (Shaughnessy *et al.*, 2001). But its activity is low. Therefore, preparing derivatives has been an active research area. Herein we report the crystal structure of the title compound (I).

In the structure of the title compound (I) (Fig.1), the S(6) ring of C(1)/C(2)/C(3)/C(4)/C(5)/C(6) in (I) is an aromatic ring. C(1)–O(1) [1.3702 (16) Å], C(6)–O(2)[1.3683 (16) Å], C(8)–O(2)[1.4181 (18) Å], and C(7)–O(3) [1.433 (2) Å] are typical for C–O single bonds.

In the crystal structure, these molecules are linked into infinite one-dimensional network by intermolecular O—H···O hydrogen bonds running along [100] (Fig. 2, Table 1).

### Experimental

4-Hydroxy-3-methoxybenzaldehyde (3.8 g, 25 mmol) was dissolved in methanol (40 ml) at 283 K. After stirring for 30 min, borohydride (0.94 g, 25 mmol) was added in reaction solution, slowly. After 4 h, the solution was quenched with water (150 ml), vacuum concentrated to remove methanol and the aqueous layer was extracted with chloroform, the combined organic extracts were washed, dried and evaporated under reduced pressure to give the crude product. Then purification by column chromatography and recrystallization from chloroform gave (I) as colourless plates (2.58 g, 67%).

### Refinement

H atoms were treated as riding, with C—H distances in the range of 0.93–0.97 Å and O—H distances of 0.85 Å, and were refined as riding with *U*_iso_(*H*)=1.2*U*_eq_(C_methylene_ and C in phenyl ring) and *U*_iso_(H)=1.5*U*_eq_(O and (C_methyl_)).

### Crystal data

**Table:**

C_8_H_10_O_3_	*F*(000) = 328
*M**_r_* = 154.16	*D*_x_ = 1.359 Mg m^−^^3^
Monoclinic, *P*2_1_/*c*	Mo *K*α radiation, λ = 0.71073 Å
Hall symbol: -P 2ybc	Cell parameters from 1768 reflections
*a* = 9.8476 (6) Å	θ = 2.6–27.6°
*b* = 6.1721 (4) Å	µ = 0.10 mm^−^^1^
*c* = 15.4915 (7) Å	*T* = 293 K
β = 126.877 (2)°	Plate, colorless
*V* = 753.19 (8) Å^3^	0.29 × 0.11 × 0.07 mm
*Z* = 4	

### Data collection

**Table:**

Bruker SMART CCD diffractometer	1475 independent reflections
Radiation source: fine-focus sealed tube	1249 reflections with *I* > 2σ(*I*)
graphite	*R*_int_ = 0.015
ω scans	θ_max_ = 26.0°, θ_min_ = 2.6°
Absorption correction: multi-scan (*SADABS*; Bruker, 2001)	*h* = −12→8
*T*_min_ = 0.971, *T*_max_ = 0.993	*k* = −7→7
3996 measured reflections	*l* = −17→19

### Refinement

**Table:**

Refinement on *F*^2^	Secondary atom site location: difference Fourier map
Least-squares matrix: full	Hydrogen site location: inferred from neighbouring sites
*R*[*F*^2^ > 2σ(*F*^2^)] = 0.037	H-atom parameters constrained
*wR*(*F*^2^) = 0.102	*w* = 1/[σ^2^(*F*_o_^2^) + (0.0462*P*)^2^ + 0.2254*P*] where *P* = (*F*_o_^2^ + 2*F*_c_^2^)/3
*S* = 1.05	(Δ/σ)_max_ < 0.001
1475 reflections	Δρ_max_ = 0.24 e Å^−^^3^
102 parameters	Δρ_min_ = −0.19 e Å^−^^3^
2 restraints	Extinction correction: *SHELXL*, Fc^*^=kFc[1+0.001xFc^2^λ^3^/sin(2θ)]^-1/4^
Primary atom site location: structure-invariant direct methods	Extinction coefficient: 0.043 (6)

### Special details

**Table:**

Geometry. All e.s.d.'s (except the e.s.d. in the dihedral angle between two l.s. planes) are estimated using the full covariance matrix. The cell e.s.d.'s are taken into account individually in the estimation of e.s.d.'s in distances, angles and torsion angles; correlations between e.s.d.'s in cell parameters are only used when they are defined by crystal symmetry. An approximate (isotropic) treatment of cell e.s.d.'s is used for estimating e.s.d.'s involving l.s. planes.
Refinement. Refinement of *F*^2^ against ALL reflections. The weighted *R*-factor *wR* and goodness of fit *S* are based on *F*^2^, conventional *R*-factors *R* are based on *F*, with *F* set to zero for negative *F*^2^. The threshold expression of *F*^2^ > σ(*F*^2^) is used only for calculating *R*-factors(gt) *etc*. and is not relevant to the choice of reflections for refinement. *R*-factors based on *F*^2^ are statistically about twice as large as those based on *F*, and *R*- factors based on ALL data will be even larger.

### Fractional atomic coordinates and isotropic or equivalent isotropic displacement parameters (Å^2^)

**Table:**

	*x*	*y*	*z*	*U*_iso_*/*U*_eq_	
O1	0.16947 (13)	0.60312 (18)	0.16129 (8)	0.0462 (3)	
H1A	0.1727	0.4901	0.1347	0.069*	
O2	0.35690 (14)	0.26017 (19)	0.27618 (8)	0.0501 (3)	
O3	0.16416 (15)	0.1981 (2)	0.52945 (9)	0.0621 (4)	
H3B	0.0675	0.2067	0.4697	0.093*	
C1	0.19570 (17)	0.5586 (2)	0.25692 (11)	0.0352 (3)	
C2	0.12924 (18)	0.6933 (2)	0.29368 (12)	0.0407 (4)	
H2A	0.0704	0.8173	0.2550	0.049*	
C3	0.14955 (19)	0.6447 (2)	0.38857 (12)	0.0416 (4)	
H3A	0.1034	0.7361	0.4125	0.050*	
C4	0.23753 (17)	0.4625 (2)	0.44737 (11)	0.0383 (3)	
C5	0.31075 (18)	0.3308 (2)	0.41213 (11)	0.0394 (4)	
H5A	0.3734	0.2100	0.4524	0.047*	
C6	0.29122 (17)	0.3781 (2)	0.31805 (11)	0.0360 (3)	
C7	0.2509 (2)	0.3978 (3)	0.54583 (12)	0.0467 (4)	
H7A	0.3696	0.3823	0.6065	0.056*	
H7B	0.2026	0.5111	0.5632	0.056*	
C8	0.4600 (2)	0.0789 (3)	0.33595 (13)	0.0491 (4)	
H8A	0.4997	0.0128	0.2987	0.074*	
H8B	0.3948	−0.0242	0.3435	0.074*	
H8C	0.5554	0.1256	0.4061	0.074*	

### Atomic displacement parameters (Å^2^)

**Table:**

	*U*^11^	*U*^22^	*U*^33^	*U*^12^	*U*^13^	*U*^23^
O1	0.0591 (7)	0.0473 (6)	0.0406 (6)	0.0118 (5)	0.0344 (5)	0.0107 (5)
O2	0.0624 (7)	0.0559 (7)	0.0461 (6)	0.0249 (5)	0.0400 (6)	0.0144 (5)
O3	0.0635 (7)	0.0802 (9)	0.0413 (6)	−0.0217 (7)	0.0308 (6)	−0.0033 (6)
C1	0.0360 (7)	0.0382 (7)	0.0338 (7)	−0.0010 (6)	0.0223 (6)	0.0021 (6)
C2	0.0446 (8)	0.0359 (8)	0.0413 (8)	0.0057 (6)	0.0256 (7)	0.0033 (6)
C3	0.0467 (8)	0.0412 (8)	0.0434 (8)	0.0016 (6)	0.0304 (7)	−0.0052 (6)
C4	0.0389 (7)	0.0440 (8)	0.0318 (7)	−0.0031 (6)	0.0212 (6)	−0.0040 (6)
C5	0.0399 (7)	0.0428 (8)	0.0339 (7)	0.0070 (6)	0.0212 (6)	0.0056 (6)
C6	0.0348 (7)	0.0403 (8)	0.0360 (7)	0.0029 (6)	0.0228 (6)	0.0008 (6)
C7	0.0542 (9)	0.0532 (10)	0.0374 (8)	−0.0009 (7)	0.0300 (7)	−0.0031 (7)
C8	0.0526 (9)	0.0500 (9)	0.0496 (9)	0.0155 (7)	0.0332 (8)	0.0079 (7)

### Geometric parameters (Å, °)

**Table:**

O1—C1	1.3702 (16)	C3—H3A	0.9300
O1—H1A	0.8200	C4—C5	1.397 (2)
O2—C6	1.3683 (16)	C4—C7	1.5041 (19)
O2—C8	1.4181 (18)	C5—C6	1.3820 (19)
O3—C7	1.433 (2)	C5—H5A	0.9300
O3—H3B	0.8416	C7—H7A	0.9700
C1—C2	1.375 (2)	C7—H7B	0.9700
C1—C6	1.3977 (19)	C8—H8A	0.9600
C2—C3	1.392 (2)	C8—H8B	0.9600
C2—H2A	0.9300	C8—H8C	0.9600
C3—C4	1.378 (2)		
			
C1—O1—H1A	109.5	C4—C5—H5A	119.6
C6—O2—C8	117.84 (11)	O2—C6—C5	125.55 (13)
C7—O3—H3B	107.5	O2—C6—C1	114.85 (12)
O1—C1—C2	119.92 (12)	C5—C6—C1	119.60 (13)
O1—C1—C6	120.39 (12)	O3—C7—C4	111.63 (12)
C2—C1—C6	119.68 (12)	O3—C7—H7A	109.3
C1—C2—C3	120.32 (13)	C4—C7—H7A	109.3
C1—C2—H2A	119.8	O3—C7—H7B	109.3
C3—C2—H2A	119.8	C4—C7—H7B	109.3
C4—C3—C2	120.58 (13)	H7A—C7—H7B	108.0
C4—C3—H3A	119.7	O2—C8—H8A	109.5
C2—C3—H3A	119.7	O2—C8—H8B	109.5
C3—C4—C5	118.93 (13)	H8A—C8—H8B	109.5
C3—C4—C7	121.65 (13)	O2—C8—H8C	109.5
C5—C4—C7	119.38 (13)	H8A—C8—H8C	109.5
C6—C5—C4	120.78 (13)	H8B—C8—H8C	109.5
C6—C5—H5A	119.6		

### Hydrogen-bond geometry (Å, °)

**Table:**

*D*—H···*A*	*D*—H	H···*A*	*D*···*A*	*D*—H···*A*
O1—H1A···O2	0.82	2.31	2.6669 (16)	107
O1—H1A···O3^i^	0.82	1.96	2.7390 (16)	158
O3—H3B···O1^ii^	0.84	2.07	2.8666 (15)	158

Symmetry codes: (i) *x*, −*y*+1/2, *z*−1/2; (ii) −*x*, *y*−1/2, −*z*+1/2.

## References

[bb1] Bruker (2001). *SAINT-Plus*, *SMART* and *SADABS* Bruker AXS, Inc., Madison, Wisconsin, USA.

[bb2] Kumar, S. S., Priyadarsini, K. I. & Sainis, K. B. (2004). *J. Agric. Food Chem.* **52**, 139–145. 10.1021/jf030319d 14709027

[bb3] Shaughnessy, D. T., Setzer, R. W. & DeMarini, D. M. (2001). *Mutat. Res.* **480–481**, 55–69. 10.1016/s0027-5107(01)00169-5 11506799

[bb4] Sheldrick, G. M. (2008). *Acta Cryst.* A**64**, 112–122. 10.1107/S0108767307043930 18156677

[bb5] Spek, A. L. (2009). *Acta Cryst* D**65**, 148–155. 10.1107/S090744490804362X PMC2631630 19171970

